# Data Modeling With Polynomial Representations and Autoregressive Time-Series Representations, and Their Connections

**DOI:** 10.1109/ACCESS.2020.3000860

**Published:** 2020-06-08

**Authors:** Asoke K. Nandi

**Affiliations:** Department of Electronic and Computer EngineeringBrunel University LondonUxbridgeUB8 3PHU.K.

**Keywords:** Data models, polynomials, autoregressive processes, time-series, signal representation, Covid-19

## Abstract

Two of the data modelling techniques - polynomial representation and time-series representation – are explored in this paper to establish their connections and differences. All theoretical studies are based on uniformly sampled data in the absence of noise. This paper proves that all data from an underlying polynomial model of finite degree $q$ can be represented perfectly by an autoregressive time-series model of order $q$ and a constant term $\mu $ as in equation (2). Furthermore, all polynomials of degree $q$ are shown to give rise to the same set of time-series coefficients of specific forms with the only possible difference being in the constant term $\mu $. It is also demonstrated that time-series with either non-integer coefficients or integer coefficients not of the aforementioned specific forms represent polynomials of infinite degree. Six numerical explorations, with both generated data and real data, including the UK data and US data on the current Covid-19 incidence, are presented to support the theoretical findings. It is shown that all polynomials of degree $q$ can be represented by an all-pole filter with $q$ repeated roots (or poles) at $z=+1$. Theoretically, all noise-free data representable by a finite order all-pole filter, whether they come from finite degree or infinite degree polynomials, can be described exactly by a finite order AR time-series; if the values of polynomial coefficients are not of special interest in any data modelling, one may use time-series representations for data modelling.

## I. Introduction

Interests in data science have been growing extremely fast in the twenty-first century. As well as interests from many different subject areas, data science is being integrated in diverse range of industries and agencies (e.g., health, transport, energy, government, society, etc.). Strictly, a time-series refers to a series of data points ordered in time. It is very common that a time-series represents data points at equally separated in time. Of course, the analytics that are created for time-series data can generally be applied to a sequence of data that are equally separated in space (e.g., images) or some other domain. There are many types of time-series models, including autoregressive models.

Although there are many types of time-series models, the earliest and an alternative way to model data is by polynomial regression. Polynomial regression models are generally fitted with the Least-squares method to obtain estimated values of the polynomial coefficients. In 1805 Legendre published the Least-squares method [1] and Gauss published it in 1809 and later in 1823 [2]. In 1815 Gergonne wrote a paper on “The application of the method of least squares to the interpolation of sequences” [3]. This is an English translation by Stigler [4] of the original paper that was written in French. In the last 120 or so years, polynomial regression contributed greatly to the development of regression analysis [5]–[7].

Although there are other ways to model data, the focus in this paper is around polynomial representation and autoregressive time-series representation. There has been a lot of research in time-series data representation [8]–[12]. For example, the main goal of time-series analysis in econometrics, geophysics, meteorology, quantitative finance, seismology, and statistics is prediction or forecasting [13]–[20]. On the other hand, it is used for signal detection and estimation in communication engineering, control engineering, and signal processing [21]–[28]. It is also used for clustering, classification, and prediction or forecasting in data mining, machine learning, and pattern recognition [29]–[34]. Mathematical modelling and time-series analysis are fundamental to many fields; a couple of very recent examples can be found in [35], [36].

In polynomial representations, observed data is a function of time (or some other variable). This function, except for the case of a constant or a straight line, represents a non-linear relationship between the time (or some other variable) and the observed data, even though the parameters are linear. On the other hand, in autoregressive (AR) time-series representation, observed data is a linear function of some of the earlier data and thus the model is linear in both data and parameters. Although both are used for data modelling, there are some fundamental differences. Hence, this paper explores many questions around polynomial and autoregressive representations with a view to establish their connections and differences. Two of these questions are:

1) Can all finite degree polynomials be expressed as finite order time series? If the answer is affirmative, what is the underlying relationship?
2) Can all finite order autoregressive time-series be represented as finite order polynomials?

This study is in the context of real-valued and uniformly sampled noise-free data. The paper presents the following original results:

1) All polynomials of degree 1 (linear), of degree 2 (quadratic), and of degree 3 (cubic) can be represented as autoregressive time-series of order 1, order 2, and order 3, with a constant respectively. This is illustrated in section II.
2) All polynomials of degree 3 can be represented by AR time-series with the set of coefficients with the same values but possibly with a different value for its constant term. This observation is also true for polynomials of degree 1 and of degree 2. This is presented in section II.
3) All polynomials of finite degree $q$ can be represented as AR time-series of order $q$ and a constant. This can be found in section III.
4) All polynomials of degree $q$ can be represented by AR time-series with one set of coefficients with the same values but possibly with a different value for its constant term. This is demonstrated in section III.
5) The corresponding time-series coefficients are integers and of specific forms, which are derived in section III.
6) Some numerical explorations from several sources of both generated data and real data, including some current Covid-19 incidence data from the UK and the US, are presented in section IV.
7) Whilst all finite degree polynomials can be represented by finite order AR time-series, the converse is not true. There are infinitely many AR time-series of finite orders that cannot be represented by finite order polynomials. Furthermore, all finite order AR time-series with either non-integer coefficients or integer coefficients not of the aforementioned specific forms represent polynomials of infinite degree. This is shown in section V.
8) Section VI shows that all polynomials of degree $q$ can be represented by an all-pole filter with $q$ repeated roots (or poles) at $z=+1$. Thus, any noise-free data representable by a finite order all-pole filter, whether they come from finite degree or infinite degree polynomials, can be described exactly by a finite order AR time-series.

## II. Method – Small Degree Polynomial

Given a set of uniformly sampled real-valued data points in discrete time, these may be represented by a polynomial or a time-series. A polynomial of degree N in continuous time can take the following form

\begin{equation*} y\left ({t }\right)= \sum \limits _{i=0}^{N} {c\left ({i }\right) t^{i}}\end{equation*}

For uniformly sampled discrete time, the continuous time, *t,* is represented as $t=nT$, where $n$ is an integer and $T$ is the sampling period. In this scenario, the above equation can be rewritten as

\begin{equation*} y\left ({nT }\right)= \sum \limits _{i=0}^{N} {c\left ({i }\right) \left ({nT }\right)^{i}}\tag{1}\end{equation*}

On the other hand, an autoregressive time-series model of order $q$, AR($q$), can be written as

\begin{equation*} y\left ({n }\right)= \sum \limits _{i=1}^{q} {a\left ({i }\right) y\left ({{n-i} }\right)+ \mu }\tag{2}\end{equation*}

and may be used to represent the set of uniformly sampled data points in discrete time.

### A. Linear Polynomial

In this subsection, an exploration of data representation by a linear polynomial and an AR time-series is carried out. For any linear polynomial, $N$ has the value of 1 in equation (1). It is easy to show from equation (1) that $y\left ({nT }\right){=}\,y\left ({{nT{-}\,T} }\right)\,+ c\left ({1 }\right) T$. By removing $T$ from indices, this can be written as $y\left ({n }\right)=y\left ({n-1 }\right)+c\left ({1 }\right) \mathrm { }T$. Comparing this with equation (2) for AR(q), it is clear that $q =1$, $a\left ({1 }\right) =1$, and $\mu =c\left ({1 }\right) T$.

Therefore, the following can be concluded:

• Every linear polynomial, i.e., of degree 1, can be perfectly represented by an AR(1) time-series.
• Every linear polynomial will have the same value of the coefficient in time-series, i.e., $a\left ({1 }\right)=1$.
• The constant term in the time-series is given by $\mu =c\left ({1 }\right) T$.
• This implies that every linear polynomial with different values of $c\left ({0 }\right)$ but the same value of $c\left ({1 }\right)$ will have the identical AR(1) representation, i.e., with the same values of $a\left ({1 }\right)$ and $\mu $.

### B. Quadratic Polynomial

In this subsection, an exploration of data representation by a quadratic polynomial and an AR time-series is carried out. For any quadratic polynomial, $N$ has the value of 2 in equation (1). Thus, it follows from equation (1) that

\begin{align*} y\left ({nT }\right)=&c\left ({0 }\right)+c\left ({1 }\right) \mathrm { }\left ({nT }\right)+c\left ({2 }\right)\left ({nT }\right)^{2}, \mathrm { }\tag{3}\\ y\left ({{nT-T} }\right)=&c\left ({0 }\right)+c\left ({1 }\right) \mathrm { }\left ({{nT-T} }\right)+c\left ({2 }\right)\left ({{nT-T} }\right)^{2}, \\ {}\tag{4}\\ y\left ({nT-2T }\right)=&c\left ({0 }\right)+c\left ({1 }\right) \mathrm { }\left ({nT-2T }\right) \\&+\, c\left ({2 }\right)\left ({nT-2T }\right)^{2},\tag{5}\end{align*}

Using equations (3) and (4), one can write

\begin{align*} y\left ({nT }\right)&=y\left ({{nT-T} }\right)+c\left ({1 }\right)T \\&\quad \qquad \qquad \qquad \qquad { +\, 2c\left ({2 }\right)nT^{2}-c\left ({2 }\right)T^{2}, } \tag{6}\end{align*}

and, using equations (4) and (5), one can write

\begin{align*} y\left ({{nT-T} }\right)&=y\left ({nT-2T }\right)+c\left ({1 }\right)T \\&\qquad \qquad \qquad\!\! \qquad { +\, 2c\left ({2 }\right)nT^{2}\!-\!3c\left ({2 }\right)T^{2}, } \!\tag{7}\end{align*}

Now, using equations (6) and (7), one finds

\begin{align*} y\left ({nT }\right)&=y\left ({{nT-T} }\right) \\&\quad { +\left [{ y\left ({{nT-T} }\right)-y\left ({nT-2T }\right) }\right]+2c\left ({2 }\right)T^{2}, } \tag{8}\end{align*}

Therefore,

\begin{equation*} y\left ({nT }\right)=2y\left ({{nT-T} }\right)- y\left ({nT-2T }\right)+2c\left ({2 }\right)T^{2}\tag{9}\end{equation*}

By removing $T$ from indices, equation (9) can be written as $y\left ({n }\right)=2y\left ({n-1 }\right)-y(n-2)+2c\left ({2 }\right)T^{2}$. Comparing this with equation (2) for AR(q), it is clear that $q =2$, $a\left ({1 }\right)=2, a\left ({2 }\right)= -1$, and $\mu =2c\left ({2 }\right)T^{2}$.

Therefore, the following can be concluded:

• Every quadratic polynomial, i.e., of degree 2, can be perfectly represented by an AR(2) time-series.
• Every quadratic polynomial will have the same coefficient values in time-series, i.e., $a\left ({1 }\right)=2$ and $a\left ({2 }\right)=-1$.
• The constant term in the time-series is given by $\mu =2c\left ({2 }\right)T^{2}$.
• This implies that every quadratic polynomial with different values of $c\left ({0 }\right)$ and $c\left ({1 }\right)$ but the same value of $c\left ({2 }\right)$ will have the identical AR(2) representation, i.e., with the same values of $a\left ({1 }\right), a\left ({2 }\right)$, and $\mu $.

### C. Cubic Polynomial

In this subsection, an exploration of data representation by a cubic polynomial and an AR time-series is carried out. For any cubic polynomial, $N$ has the value of 3 in equation (1). Thus, it follows from equation (1) that

\begin{align*} y\left ({nT }\right)=&c\left ({0 }\right)+c\left ({1 }\right) \mathrm { }\left ({nT }\right) \\&+ c\left ({2 }\right)\left ({nT }\right)^{2}+c\left ({3 }\right)\left ({nT }\right)^{3}, \tag{10}\\ y\left ({{nT-T} }\right)=&c\left ({0 }\right)+c\left ({1 }\right) \mathrm { }\left ({{nT-T} }\right) \\&+\, c\left ({2 }\right)\left ({nT\!-\!T }\right)^{2}\!+\! c\left ({3 }\right)\left ({nT-T }\right)^{3},\qquad \tag{11}\\ y\left ({nT-2T }\right)=&c\left ({0 }\right)+c\left ({1 }\right) \mathrm { }\left ({nT-2T }\right) \\&+\, c\left ({2 }\right)\left ({nT-2T }\right)^{2}+ c\left ({3 }\right)\left ({nT-2T }\right)^{3}, \\ {}\tag{12}\\ y\left ({nT-3T }\right)=&c\left ({0 }\right)+c\left ({1 }\right) \mathrm { }\left ({nT-3T }\right) \\&+\, c\left ({2 }\right)\left ({nT-3T }\right)^{2}+ c\left ({3 }\right)\left ({nT-3T }\right)^{3}, \\ {}\tag{13}\end{align*}

Using equations (10) and (11), one can obtain

\begin{align*} y\left ({nT }\right)=&y\left ({{nT-T} }\right)+c\left ({1 }\right)T+c\left ({2 }\right)2nT^{2} \\&-\, c\left ({2 }\right)T^{2}+c\left ({3 }\right)3n^{2}T^{3}-c\left ({3 }\right)3nT^{3}+c\left ({3 }\right)T^{3}, \\ {}\tag{14}\end{align*}

and, using equations (11) and (12), one can obtain

\begin{align*} y\left ({{nT-T} }\right)=&y\left ({nT-2T }\right)+c\left ({1 }\right)T \\&+\, c\left ({2 }\right)2nT^{2}-c\left ({2 }\right)3T^{2}+c\left ({3 }\right)3n^{2}T^{3} \\&-\, c\left ({3 }\right)9nT^{3}+c\left ({3 }\right)7T^{3},\tag{15}\end{align*}

Now, using equations (14) and (15), one obtains

\begin{align*} y\left ({nT }\right) \!- \!y\left ({{nT-T} }\right)=&y\left ({nT-T }\right)-y\left ({nT-2T }\right) \\&+\, 2c\left ({2 }\right)T^{2}\!+6c\left ({3 }\right)nT^{3}\!-6c\left ({3 }\right)T^{3}, \\ {}\tag{16}\end{align*}

Using equations (12) and (13), one can write

\begin{align*} y\left ({nT-2T }\right)=&y\left ({nT-3T }\right)+c\left ({1 }\right)T+c\left ({2 }\right)2nT^{2} \\&- \,c\left ({2 }\right)5T^{2}+c\left ({3 }\right)3n^{2}T^{3} \\&-\,c\left ({3 }\right)15nT^{3}+19c\left ({3 }\right)T^{3},\tag{17}\end{align*}

Now, using equations (15) and (17), one can write

\begin{align*}&\hspace {-2pc} y\left ({{nT-T} }\right) - y\left ({nT-2T }\right) \\=&y\left ({nT-2T }\right)- y\left ({nT-3T }\right)+2c\left ({2 }\right)T^{2} \\&+\,6c\left ({3 }\right)nT^{3}-12c\left ({3 }\right)T^{3},\tag{18}\end{align*}

Thus,

\begin{align*}& y\left ({{nT-T} }\right) - 2y\left ({nT-2T }\right)+y\left ({nT-3T }\right) \\&\quad { +\, 6c\left ({3 }\right)T^{3}=2c\left ({2 }\right)T^{2}+6c\left ({3 }\right)nT^{3}-6c\left ({3 }\right)T^{3}, } \tag{19}\end{align*}

Combining equations (16) and (19), one obtains

\begin{align*} y\left ({nT }\right) - y\left ({{nT-T} }\right)=&y\left ({nT-T }\right)-y\left ({nT-2T }\right) \\&+\,y\left ({{nT-T} }\right) - \mathrm { }2y\left ({nT-2T }\right) \\&+\,y\left ({nT-3T }\right)+6c\left ({3 }\right)T^{3},\end{align*}

Therefore,

\begin{align*} y\left ({nT }\right)&=3y\left ({{nT-T} }\right)- 3y\left ({nT-2T }\right) \\& \qquad \qquad \qquad \qquad { +\, y\left ({nT-3T }\right)+6c\left ({3 }\right)T^{3} } \tag{20}\end{align*}

By removing $T$ from indices, this can be written as $y\left ({n }\right)=3y\left ({n-1 }\right)-3y\left ({n-2 }\right)+y\left ({n-1 }\right)+6c\left ({3 }\right)T^{3}$. This can be described by AR($q$), provided $q =3$, $a\left ({1 }\right)=3, a\left ({2 }\right)= -3, a\left ({3 }\right)=1$, and $\mu =6c\left ({3 }\right)T^{3}$.

Therefore, the following can be concluded:

• Every cubic polynomial, i.e., of degree 3, can be perfectly represented by an AR(3) time-series.
• All cubic polynomials will have the same coefficient values in time-series, i.e., $a\left ({1 }\right)=3$, $a\left ({2 }\right)=-3$, and $a\left ({3 }\right)=1$.
• The constant term in the time-series is given by $\mu =6c\left ({3 }\right)T^{3}$.
• This implies that every cubic polynomial with different values of $c\left ({0 }\right)$, $c\left ({1 }\right)$, and $c\left ({2 }\right)$ but the same value of $c\left ({3 }\right)$ will have the identical AR(3) representation, i.e., with the same values of $a\left ({1 }\right), a\left ({2 }\right)$, $a\left ({3 }\right), $ and $\mu $.

The summary of the exposition so far is that all polynomials of degree 1 (linear), of degree 2 (quadratic), and of degree 3 (cubic) can be perfectly represented as AR time-series of orders 1, 2, and 3 respectively. Furthermore, for each degree of polynomials all the time-series coefficients have predefined values and they are specific integers, while the constant term, $\mu $, has a predefined form that depends on the coefficient of the leading degree of the polynomial, the degree of the polynomial, and the sampling period. These and more specific information can be found in Table 1 above.

**TABLE 1:** Information for Polynomials of Different Degrees

Degree of polynomial $N$	AR parameters			
	$a\left ({1 }\right)$	$a\left ({2 }\right)$	$a\left ({3 }\right)$	$\mu $
1	1			$c\left ({1 }\right)T$
2	2	−1		$2c\left ({2 }\right)T^{2}$
3	3	−3	1	$6c\left ({3 }\right)T^{3}$

## III. Method – Any Finite Degree Polynomial

In section II it has been demonstrated that all polynomials of degree 1 (linear), of degree 2 (quadratic), and of degree 3 (cubic) can be perfectly represented as autoregressive time-series of orders 1, 2, and 3 respectively. In this section the exploration is generalised for all polynomials of every finite degree. In section II it was found that, for $q=1, 2, $ and 3, the degree of the polynomial and the corresponding order of the AR time-series order are identical. In the following, a discrete-time polynomial of degree $q$ of the form below is considered

\begin{equation*} y\left ({nT }\right)= \sum \limits _{j=0}^{q} {c\left ({j }\right) \left ({nT }\right)^{j}}\tag{21}\end{equation*}

in seeking a corresponding autoregressive time-series model of order $q$, AR($q$).

The time-series in equation (2) can be rewritten as

\begin{equation*} y\left ({n }\right)- \mu = \sum \limits _{i=1}^{q} {a\left ({i }\right) y\left ({{n-i} }\right)}\tag{22}\end{equation*}

Now it is conjectured that

\begin{align*} a\left ({i }\right)=\left ({-1 }\right)^{i+1} \left({\begin{array}{c}q\\ i \end{array}}\right)\tag{23}\end{align*}

for $i=1, 2, \ldots, \mathrm { }q$. Using this conjecture and equation (21), the equation (22) can be written as

\begin{align*} y\left ({nT }\right)-\mu = \sum \limits _{i=1}^{q} {\left ({-1 }\right)^{i+1}\left({\begin{array}{c}q\\ i \end{array}}\right)\sum \limits _{j=0}^{q} {c\left ({j }\right) \left ({{nT-iT} }\right)^{j}}}\tag{24}\end{align*}

In the above double summation, it is instructive and revealing to consider different values of $j$ separately.

### A. Part I

For the particular case of $j=0$, the right-hand side of equation (24) can be written as $\sum \nolimits _{i=1}^{q} {\left ({-1 }\right)^{i+1}\binom {q}{i} c(0)} $. The relation 0.154.6 on page 4 of [37], for $q\ge n\ge 1$ and ${0}^{0} \equiv 1$, can be adapted to

\begin{align*} \sum \limits _{i=0}^{q} {\left ({-1 }\right)^{i}\left({\begin{array}{c}q\\ i \end{array}}\right)} i^{n-1} =0\tag{25}\end{align*}

Using this relation, for $q\ge n=1$,

\begin{align*} \sum \limits _{i=1}^{q} {\left ({-1 }\right)^{i+1}\left({\begin{array}{c}q\\ i \end{array}}\right)}=&\sum \limits _{i=0}^{q} {\left ({-1 }\right)^{i+1}\left({\begin{array}{c}q\\ i \end{array}}\right)} +1 \\=&-\sum \limits _{i=0}^{q} {\left ({-1 }\right)^{i}\left({\begin{array}{c}q\\ i \end{array}}\right)} +1=1\tag{26}\end{align*}

Therefore, for the case of $j=0$, the right-hand side of equation (24) is $c\left ({0 }\right)$.

### B. Part II

For the case of $j=1$, the right-hand side of equation (24) can be written as

\begin{align*}&\hspace {-2pc}\sum \nolimits _{i=1}^{q} {\left ({-1 }\right)^{i+1}\left({\begin{array}{c}q\\ i \end{array}}\right) \left \{{c\left ({1 }\right) \left ({{nT-iT} }\right) }\right \}} \\&= c\left ({1 }\right)nT \sum \nolimits _{i=1}^{q} \left ({-1 }\right)^{i+i}\left({\begin{array}{c}q\\ i \end{array}}\right) \\&-\,c\left ({1 }\right)T \sum \nolimits _{i=1}^{q} {\left ({-1 }\right)^{i+1}\mathrm { }\left({\begin{array}{c}q\\ i \end{array}}\right)} i\tag{27}\end{align*}

Using equation (25), for $q\ge n>1$, one can write

\begin{align*} \sum \limits _{i=1}^{q} {\left ({-1 }\right)^{i+1}\left({\begin{array}{c}q\\ i \end{array}}\right)} i^{n-1}=&\sum \limits _{i=0}^{q} {\left ({-1 }\right)^{i+1}\mathrm { }\left({\begin{array}{c}q\\ i \end{array}}\right)} i^{n-1} \\=&-\sum \limits _{i=0}^{q} {\left ({-1 }\right)^{i}\left({\begin{array}{c}q\\ i \end{array}}\right)} i^{n-1}=0\tag{28}\end{align*}

Using equations (26) and (28) in equation (27), it is found that the right-hand side of equation (24), for the case of $j=1$, is equal to $c\left ({1 }\right)nT$.

### C. Part III

Now the case of $j=2$ is considered. The right-hand side of equation (24) can be written as

\begin{align*}&\hspace {-2pc}\sum \limits _{i=1}^{q} {\left ({-1 }\right)^{i+1}\left({\begin{array}{c}q\\ i \end{array}}\right) \left \{{c\left ({2 }\right) \left ({nT-iT }\right)^{2} }\right \}} \\=&c\left ({2 }\right)\left ({nT }\right)^{2} \sum \limits _{i=1}^{q} \left ({-1 }\right)^{i+i}\left({\begin{array}{c}q\\ i \end{array}}\right) \\&-\,2c\left ({2 }\right)nT^{2}\sum \limits _{i=1}^{q} {\left ({-1 }\right)^{i+1}\left({\begin{array}{c}q\\ i \end{array}}\right)} i \\&+\,c\left ({2 }\right){(-T)}^{2} \sum \limits _{i=1}^{q} {\left ({-1 }\right)^{i+1}\left({\begin{array}{c}q\\ i \end{array}}\right)} i^{2}\end{align*}

Using equations (26) and (28), in the previous expression for the right-hand side of equation (24), for the case of $j=2$, the right-hand side is found to be equal to $c\left ({2 }\right) \mathrm { }\left ({nT }\right)^{2}$.

Similarly, for each value of $j$ up to $j=q-1$, it can be shown that the right-hand side of equation is equal to $c\left ({j }\right) \left ({nT }\right)^{j}$. When $j=q-1$, there is a term of the form $c\left ({q-1 }\right){(-T)}^{q-1} \sum \nolimits _{i=1}^{q} {\left ({-1 }\right)^{i+1}\binom {q}{i} } \mathrm { }i^{q-1}$. According to equation (28), which is valid if the top range of the summation is either larger than or equal to the power of $i$ plus one, i.e., $q\ge \left ({q-1 }\right)+1$, this term equates to zero.

However, when $j=q$, there is a term of the form $c\left ({q }\right)(-{T})^{q} \sum \nolimits _{i=1}^{q} {\left ({-1 }\right)^{i+1}\binom {q}{i}} i^{q}$. For this term, equation (28) is not valid since the top range of the summation, i.e., $q$, is neither larger than nor equal to the power of $i$ plus one, i.e., $\left ({q+1 }\right)$. To deal with the case of $j=q$, the relation 0.154.4 on page 4 of [37], for $n\ge 0$ and ${0}^{0} \equiv 1$, can be adapted to

\begin{align*} \sum \limits _{i=0}^{q} {\left ({-1 }\right)^{i}\left({\begin{array}{c}q\\ i \end{array}}\right)} i^{q}=\left ({-1 }\right)^{q}q!\tag{29}\end{align*}

Thus,

\begin{align*} \sum \limits _{i=1}^{q} {\left ({-1 }\right)^{i+1}\left({\begin{array}{c}q\\ i \end{array}}\right)} i^{q}=&\sum \limits _{i=0}^{q} {\left ({-1 }\right)^{i+1}\left({\begin{array}{c}q\\ i \end{array}}\right)} i^{q} \\=&-\sum \limits _{i=0}^{q} {\left ({-1 }\right)^{i}\left({\begin{array}{c}q\\ i \end{array}}\right)} i^{q}=-\left ({-1 }\right)^{q}q! \\ {}\tag{30}\end{align*}

Thus, for the case of $j=q$, the right-hand side of equation (24) can be written as

\begin{align*}&\hspace {-1.2pc} \sum \limits _{i=1}^{q} {\left ({-1 }\right)^{i+1}\left({\begin{array}{c}q\\ i \end{array}}\right) \left \{{c\left ({q }\right) \left ({nT-iT }\right)^{q} }\right \}} \\=&c\left ({q }\right)\left ({nT }\right)^{q} \sum \limits _{i=1}^{q} \left ({-1 }\right)^{i+i}\left({\begin{array}{c}q\\ i \end{array}}\right)+\ldots +c\left ({q }\right)(-{T)}^{q} \\&\times \,\sum \limits _{i=1}^{q} {\left ({-1 }\right)^{i+1}\left({\begin{array}{c}q\\ i \end{array}}\right)} i^{q}\end{align*}

Using equation (26) the first term is $c\left ({q }\right)\left ({nT }\right)^{q}$. All the terms in the middle are zero by virtue of equation (28). Using equation (30) the last term is found to be $c\left ({q }\right)\left ({-T }\right)^{q} \left ({-\left ({-1 }\right)^{q}q! }\right)$, which is equal to $-c\left ({q }\right)T^{q}(q!)$.

Adding all the results for $j=0, 1, \mathrm { }\ldots, q$, one obtains

\begin{align*} y\left ({nT }\right)-\mu=&\sum \limits _{j=0}^{q} {c\left ({j }\right) \mathrm { }\left ({nT }\right)^{j}} - c\left ({q }\right)T^{q}\left ({q! }\right) \\=&y\left ({nT }\right)- c\left ({q }\right)T^{q}\left ({q! }\right)\end{align*}

Thus,

\begin{equation*} \mu =c\left ({q }\right)T^{q}\left ({q! }\right)\tag{31}\end{equation*}

Therefore, all noise-free data from uniformly sampled polynomials of finite degree $q$ can be perfectly represented by an autoregressive time-series model of order $q$ such that

\begin{equation*} y\left ({n }\right)= \sum \limits _{i=1}^{q} {a\left ({i }\right) \mathrm { }y\left ({{n-i} }\right)+ \mu }\end{equation*}

where

\begin{equation*} \mu =c\left ({q }\right)T^{q}\left ({q! }\right)\end{equation*}

and

\begin{align*} a\left ({i }\right)=\left ({-1 }\right)^{i+1} \left({\begin{array}{c}q\\ i \end{array}}\right),\quad \text {for}~{i} = 1, 2, \ldots, q\end{align*}

## IV. Experiments

In this section some explorations are carried out for different types of data sources to illustrate a few themes. In reality, all real data have uncertainties; therefore, it is important to study sensitivities to degrees and types of uncertainties. Yet, in these explorations all generated data are error-free. Here the objectives are to underpin some theoretical results and to generate some intuitions from precise data and theoretical results, and not to get distracted into studying effects of noise interference. Two applications to real data, the current Covid-19 data from the UK and the US, are clearly not noise-free but are offered as real examples.

In the first four of these explorations, N data are generated. These are then modelled by polynomials as in equation (21) and time-series as in equation (2). When considering a polynomial of degree $p$, the first $(p+1)$ data are used to evaluate the $(p+1)$ coefficients of this polynomial. This works as data are error-free. On the other hand, when considering a time-series of order $q$, the first $2q$ data are used to evaluate the $q$ coefficients of this time-series.

### A. Case I

Here $N$ data are generated from a polynomial of degree 3,

\begin{align*} y\left ({n }\right)&=(n{T)}^{3}-2\left ({nT }\right)^{2} \\&\qquad { +\left ({nT }\right)-1, ~\text {for} ~T=0.2\quad \text {and} ~n=-17:1:17. } \end{align*}

This is a finite degree polynomial with no steady state. For each value of the degree $p$ of the polynomial from $p=1, \mathrm { }\ldots, 9$, the first $(p+1)$ data are used to calculate the $(p+1)$ coefficients of the polynomial. Using these polynomial coefficients, the remaining $(N-p-1)$ data values are predicted; these are labelled as $yp(n)$ for $n=(p+2), \mathrm { }\ldots, N$. Similarly, for each value of the time-series order of $q$ from $q=1, \ldots, 9$, the first $2q$ data are used to calculate the $q$ coefficients of the time-series. Using these coefficients, the remaining $(N-2q)$ data values are predicted; these are labelled as $yt(n)$ for $n=(2q+1), \ldots, \mathrm { }N$.

For the same values of $(p+1)$ and $q$, $(N-p-1)$ data values are predicted for polynomial and $(N-2q)$ data values are predicted for time-series representations. To compare prediction errors from polynomial and time-series representations fairly, only those predictions, i.e., $(N-2q)$ data values, common to both representations are used. Mean prediction errors are $\left ({\sum \nolimits _{i=2q+1}^{N} \left ({yp\left ({i }\right)- y\left ({i }\right) }\right) }\right)/ (N-2q)$ and $\left ({\sum \nolimits _{i=2q+1}^{N} \left ({yt\left ({i }\right)- y\left ({i }\right) }\right) }\right)/(N-2q)$ for polynomial and time-series respectively. Also, the RMS prediction error (polynomial) is defined as

\begin{equation*} \sqrt {\left [{ \left ({\sum \nolimits _{i=2q+1}^{N} \left ({yp\left ({i }\right)- y\left ({i }\right) }\right)^{2} }\right)/(N-2q) }\right]}\end{equation*}

while the RMS prediction error (time-series) is defined as

\begin{equation*} \sqrt {\left [{ \left ({\sum \nolimits _{i=2q+1}^{N} \left ({yt\left ({i }\right)- y\left ({i }\right) }\right)^{2} }\right)/(N-2q) }\right]}\end{equation*}

**FIGURE 1.**
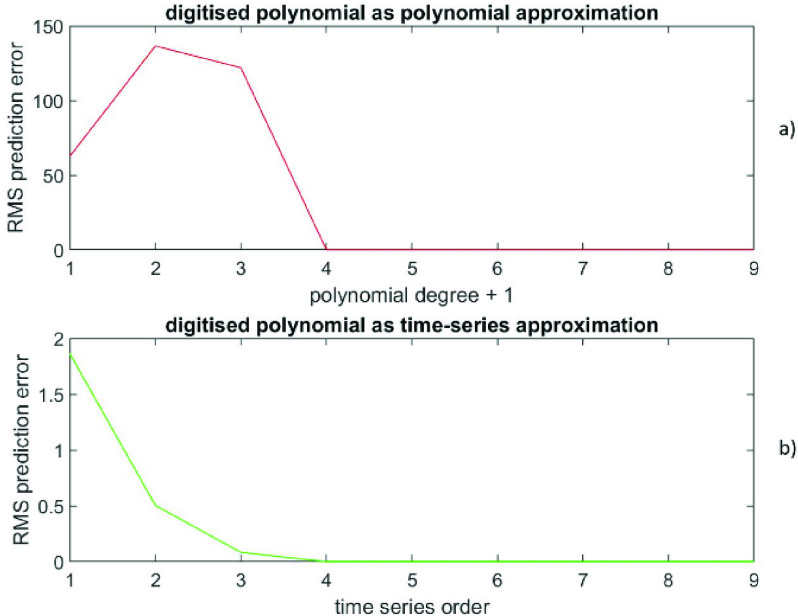
Data generated from a polynomial, Figure 1a) shows the RMS prediction error (polynomial) as a function of ($p+1$). Figure 1b) presents the RMS prediction error (time-series) as a function of $q$.

**FIGURE 2.**
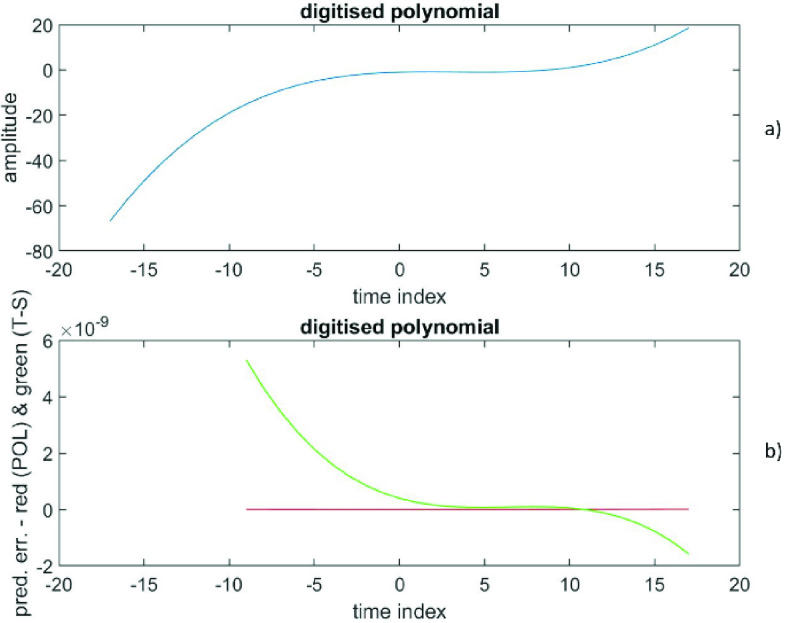
Data generated from a polynomial, Figure 2a) depicts the data versus the time index. Figure 2b) shows the prediction errors versus the time index for ($p+1) = 4$ (polynomial in red) and for $q = 4$ (time-series in green).

The RMS prediction error (polynomial) is depicted in Figure 1a) as a function of $(p+1)$, while the RMS prediction error (time-series) is shown in Figure 1b) as a function of $q$. The prediction error at $\left ({p+1 }\right)=4$ is (6.6 * 10^−13^ ± 2.7 * 10^−12^), while the prediction error at $q=4$ is (7.2 * 10^−10^ ± 1.6 * 10^−9^); both are extremely small. Figure 2a) shows the data versus the time index, while the Figure 2b) depicts the prediction errors versus the time index for $\left ({p+1 }\right)=4$ (polynomial in red) and at $q=4$ (time-series in green). The results confirm that these data from a finite degree polynomial can be equally well described by both polynomial and time-series representations.

### B. Case II

Here $N$ data are generated from a sine wave

\begin{equation*} y\left ({n }\right)=sin(2\pi n/16), \quad \text {for} ~n=-17:1:17.\end{equation*}

This represents an infinite degree polynomial and has no steady state, but its values are bounded between −1 and +1. The procedures for calculating the $(p+1)$ coefficients of the polynomial and calculating the $q$ coefficients of the AR time-series are the same as described in Case I earlier. Also, the procedures for calculating the prediction error (polynomial) and the prediction error (times-series) have been described earlier in Case I.

**FIGURE 3.**
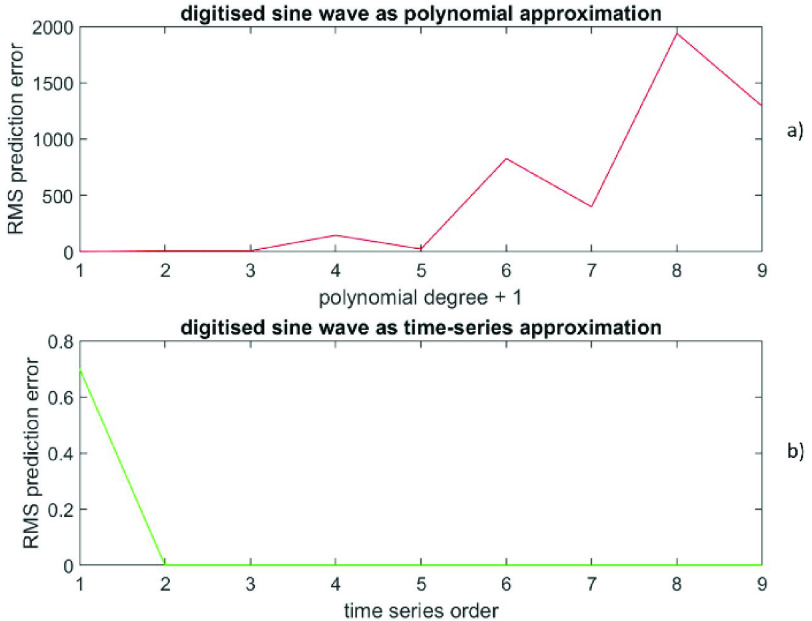
Data generated from a sine wave, Figure 3a) presents the RMS prediction error (polynomial) as a function of ($p+1$). Figure 3b) displays the RMS prediction error (time-series) as a function of $q$.

**FIGURE 4.**
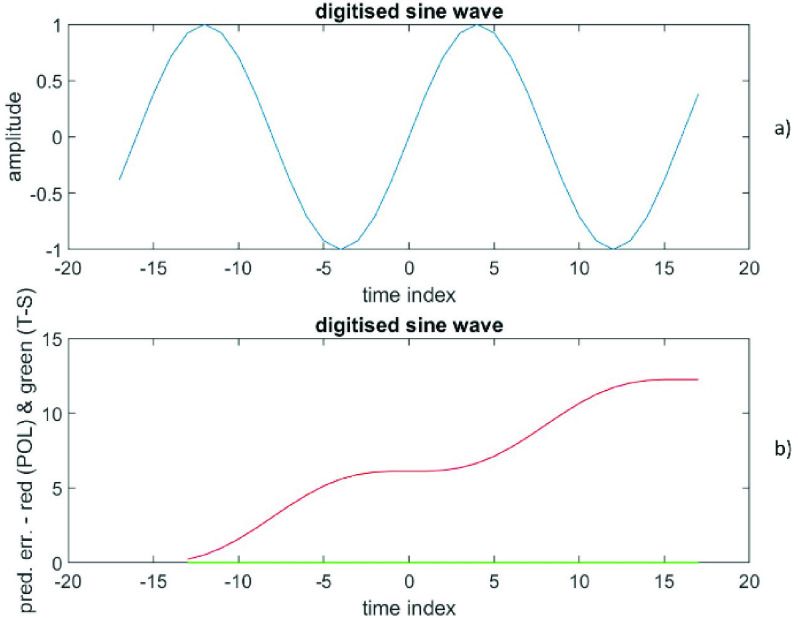
Data generated from a sine wave, Figure 4a) shows the data versus the time index. Figure 4b) depicts the prediction errors versus the time index for ($p+1) = 1$ (polynomial in red) and for $q = 2$ (time-series in green).

The RMS prediction error (polynomial) is depicted in Figure 3a) as a function of $(p+1)$, while the RMS prediction error (time-series) is shown in Figure 3b) as a function of $q$. The error at $\left ({p+1 }\right)=1$ is (6.9 ± 3.8), while the error at $q=2$ is (2.5 * 10^−18^ ± 1.4 * 10^−15^). Also, the error at $\left ({p+1 }\right)=6$ is (422 ± 614), while the error at $q=2$ is (1.3 * 10^−16^ ± 1.4 * 10^−15^). Figure 4a) shows the data versus the time index, while the Figure 4b) depicts the prediction errors versus the time index for $\left ({p+1 }\right)=1$ (polynomial in red) and at $q=2$ (time-series in green). The results confirm that these data from a sine wave are extremely well described by a time-series representation of only order 2; there is a theoretical reason for this (see section V for an explanation). Also, this time series representation is far better than any finite degree polynomial representation.

### C. Case III

Here $N$ data are generated from a non-polynomial

\begin{equation*} y\left ({n }\right)\!=\!1-2n\!-\!3n\left ({n-1 }\right)\!+\!\left ({0.5 }\right)^{n}, \quad \text {for}~n\!=\!-17:1:17.\end{equation*}

This represents an infinite degree polynomial and has no steady state. The procedures for calculating the $(p+1)$ coefficients of the polynomial and calculating the $q$ coefficients of the time-series are the same as described in Case I earlier. Also, the procedures for calculating the prediction error (polynomial) and the prediction error (times-series) have been described earlier in Case I.

**FIGURE 5.**
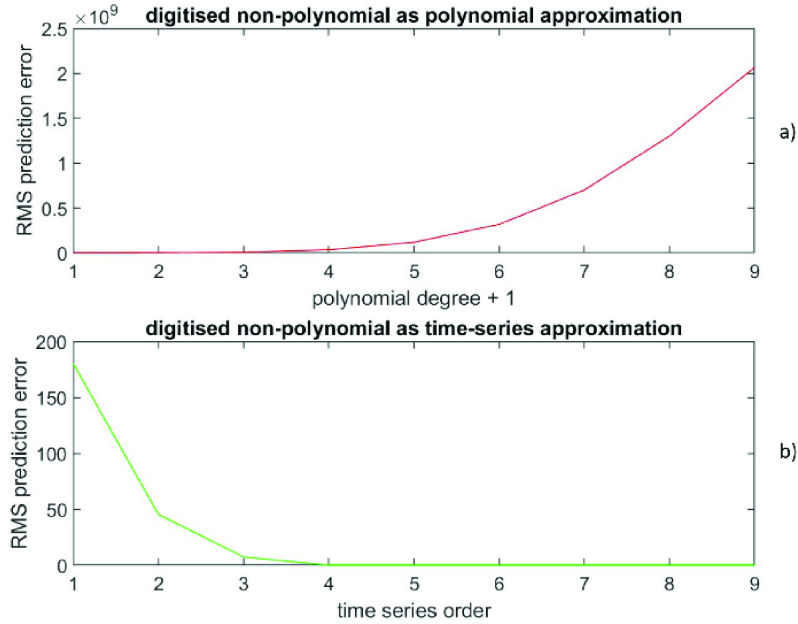
Data generated from a non-polynomial, Figure 5a) presents the RMS prediction error (polynomial) as a function of ($p+1$). Figure 5b) displays the RMS prediction error (time-series) as a function of $q$.

**FIGURE 6.**
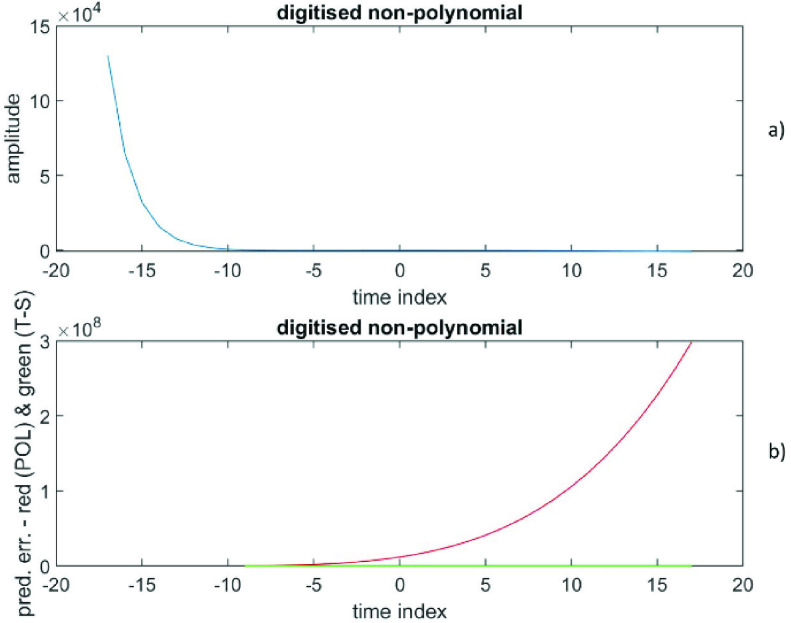
Data generated from a non-polynomial, Figure 6a) shows the data versus the time index. Figure 6b) depicts the prediction errors versus the time index for ($p+1) = 5$ (polynomial in red) and for $q = 4$ (time-series in green).

The RMS prediction error (polynomial) is depicted in Figure 5a) as a function of $(p+1)$, while the RMS prediction error (time-series) is shown in Figure 5b) as a function of $q$. The prediction error at $\left ({p+1 }\right)=3$ is (6.6 * 10^6^ ± 4.9 * 10^6^), while the prediction error at $q=4$ is (−1.2 * 10^−8^ ± 8.7 * 10^−8^). Also, the prediction error at $\left ({p+1 }\right)=5$ is (7.3 * 10^7^ ± 8.9 * 10^7^), while the prediction error at $q=4$ is (−1.2 * 10^−8^ ± 8.7 * 10^−8^). Figure 6a) shows the data versus the time index, while the Figure 6b) depicts the prediction errors versus the time index for $\left ({p+1 }\right)=5$ (polynomial in red) and at $q=4$ (time-series in green). Thus, these data from a non-polynomial are significantly better described by a time-series representation of only order 4; the theoretical reason can be found in section V. Also, RMS (time-series) is many orders of magnitude smaller than RMS (any finite degree polynomial).

### D. Case IV

Here $N$ data are generated from an inverse polynomial

\begin{equation*} y\left ({n }\right)=1/(1+0.2n), \quad \text {for}~n=0:1:34.\end{equation*}

This represents an infinite degree polynomial. It has neither a finite degree polynomial representation nor a finite order time-series representation. The procedures for calculating the $(p+1)$ coefficients of the polynomial and calculating the $q$ coefficients of the time-series are the same as described in Case I earlier. Also, the procedures for calculating the prediction error (polynomial) and the prediction error (times-series) have been described earlier in Case I.

**FIGURE 7.**
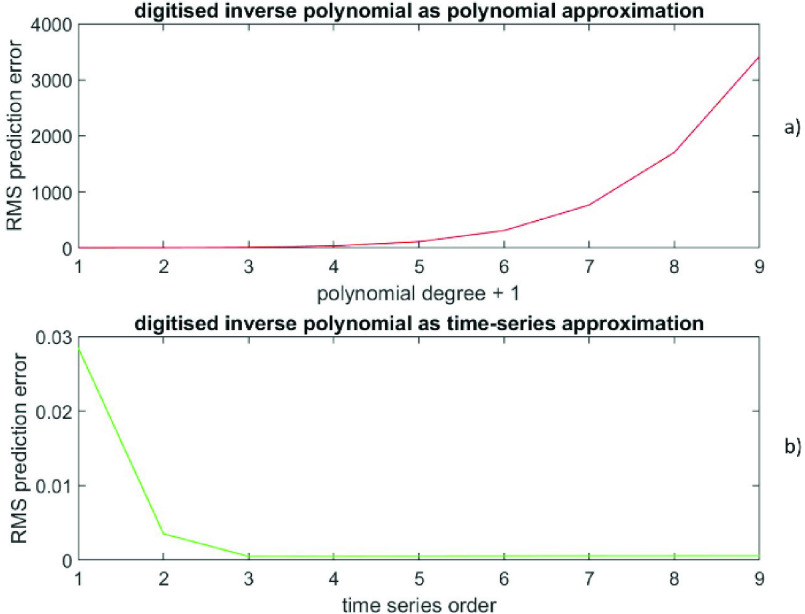
Data generated from an inverse polynomial, Figure 7a) displays the RMS prediction error (polynomial) as a function of ($p+1$). Figure 7b) presents the RMS prediction error (time-series) as a function of $q$.

**FIGURE 8.**
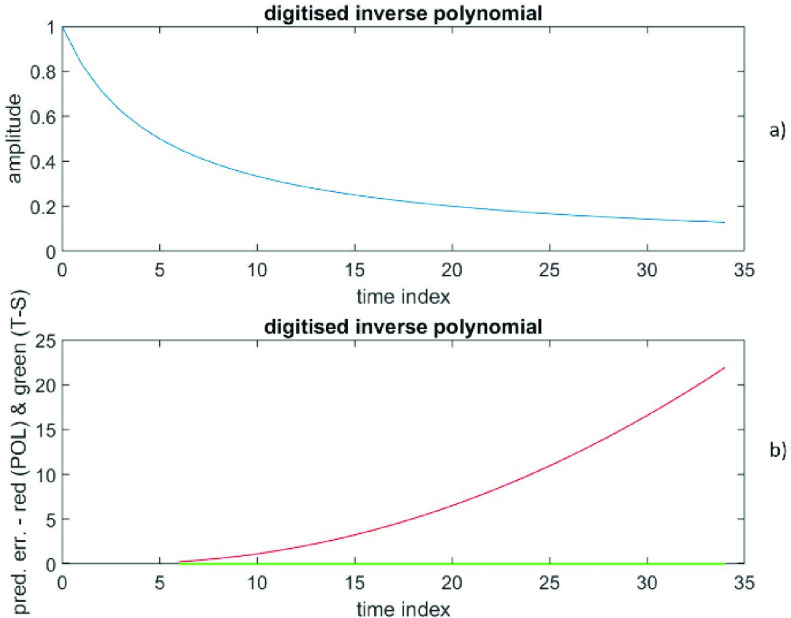
Data generated from an inverse polynomial, Figure 8a) shows the data versus the time index. Figure 8b) depicts the prediction errors versus the time index for ($p+1) = 3$ (polynomial in red) and for $q = 3$ (time-series in green).

The RMS prediction error (polynomial) is depicted in Figure 7a) as a function of $(p+1)$, while the RMS prediction error (time-series) is shown in Figure 7b) as a function of $q$. The prediction error at $\left ({p+1 }\right)=3$ is (8.2 ± 6.7), while the prediction error at $q=3$ is (−4.7 * 10^−4^ ± 1.7 * 10^−4^). Also, the prediction error at $\left ({p+1 }\right)=7$ is (476 ± 617), while the prediction error at $q=7$ is (−1.1 * 10^−7^ ± 9.5 * 10^−8^). Figure 8a) shows the data versus the time index, while the Figure 8b) depicts the prediction errors versus the time index for $\left ({p+1 }\right)=3$ (polynomial in red) and at $q=3$ (time-series in green). Results confirm that these data from an inverse polynomial are significantly better described by an AR time-series representation than a finite degree polynomial representation by several orders of magnitude in RMS.

### E. Case V

This is an example of using real data from a current global Covid-19 epidemic as it is unfolding. The dataset represents cumulative daily confirmed cases of Covid-19 infections in the UK. This dataset is publicly available [38]. On 01 April 2020 there were 61 data (i.e., N = 61) covering the period from 31 January 2020 to 31 March 2020. Thus $y(n)$ for $n=1:1:61$ represents the cumulative daily confirmed cases of Covid-19 infections in the UK.

Of these 61 data, the first 50 data are used for estimating the free parameters and the last 11 data are used for forecasting. For a polynomial of the degree $p$, the first 50 data are used to estimate the $\left ({p+1 }\right)$ coefficients of the polynomial using the Moore-Penrose inverse. By adopting the equation (21), the first 50 data can be described the matrix equation $Y=ACp$, where

\begin{align*} Y=&\left [{ y\left ({1 }\right) y\left ({2 }\right)\ldots y\left ({50 }\right) }\right]^{T} \\ Cp=&\left [{ c\left ({0 }\right) c\left ({1 }\right)\ldots c\left ({p }\right) }\right]^{T} \\ A=&[1 1\ldots 1;1 2\ldots 2^{p};\ldots;1 50\ldots {50}^{p}]\end{align*}

Thus, $Y$ is a column vector of size $50\times 1$, $C$ is a column vector of size $(p+1)$*x1*, and $A$ is a matrix of size *50x*$(p+1)$. Now,

\begin{equation*} Cp=\left ({A^{T}A }\right)^{-1}A^{T}Y\tag{32}\end{equation*}

Using these estimated polynomial coefficients from the equation (32), all 61 data are calculated using

\begin{equation*} Yp=BCp\tag{33}\end{equation*}

where $B$ is a matrix of size *61x* $(p+1)$ and $B=[1 1\ldots 1;1 2\ldots 2^{p};\ldots;1 61\ldots {61}^{p}]$, while *Yp* is a column vector of size $61\times 1$ and $Yp=\left [{ yp\left ({1 }\right) yp\left ({2 }\right)\ldots yp\left ({61 }\right) }\right]^{T}$. Of course, $\left [{ yp\left ({1 }\right) \mathrm { }yp\left ({2 }\right)\ldots yp\left ({50 }\right) }\right]^{T}$ came from the regression but $\left [{ yp\left ({51 }\right) yp\left ({52 }\right)\ldots yp\left ({61 }\right) }\right]^{T}$ are polynomial predictions for $\left [{ y\left ({51 }\right) y\left ({52 }\right)\ldots y\left ({61 }\right) }\right]^{T}$.

Similarly, for a time-series of order $q$, the first 50 data are used to estimate the $q$ coefficients of the time-series. Each of these data values depends on the coefficients and earlier data values. As all data values are error prone, the Total Least Squares, which takes account of errors in both the dependent and independent variables, is more appropriate than the ordinary Least Squares, which takes account of only errors in dependent variables and not in the independent variables. Using the $q$ estimated coefficients, all 61 data are calculated, which are labelled as $YT=\left [{ yt\left ({1 }\right) yt\left ({2 }\right)\ldots yt\left ({61 }\right) }\right]^{T}$. Of course, out of these 61 values, $\left [{ yt\left ({1 }\right) yt\left ({2 }\right)\ldots yt\left ({50 }\right) }\right]^{T}$ came from the regression and $\left [{ yt\left ({51 }\right) yt\left ({52 }\right)\ldots yt\left ({61 }\right) }\right]^{T}$ are time-series predictions for $\left [{ y\left ({51 }\right) y\left ({52 }\right)\ldots y\left ({61 }\right) }\right]^{T}$.

**FIGURE 9.**
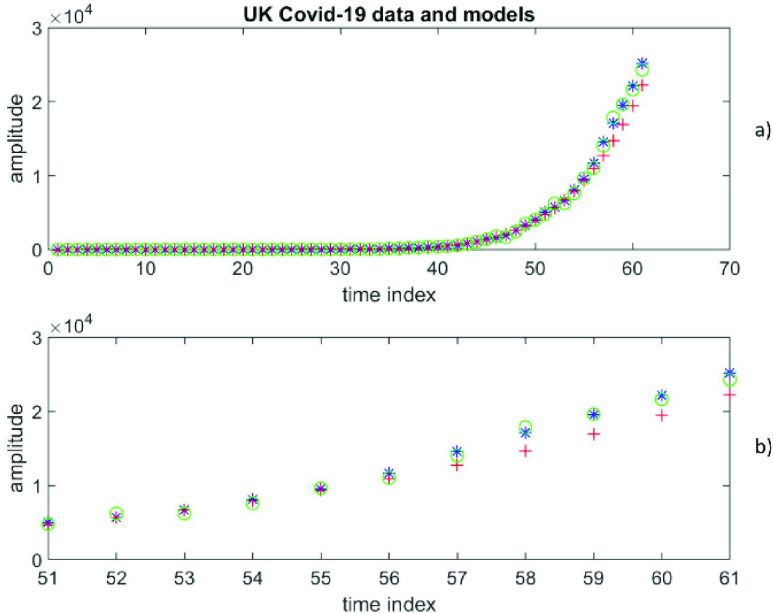
Daily confirmed cases of Covid-19 infections in the UK, covering the period from 31 January 2020 to 31 March 2020, Figure 9a) depicts all 61 data values (y) in blue, all 61 calculated values (*yp*) in red according to polynomial representation at ($p+1) = 6$ [the first 50 values are from the fit and the last 11 values are predictions], as well as all 61 calculated values (*yt*) in green according to autoregressive time-series representation of order 2 [the first 50 values are from the fit and the last 11 values are predictions]. Figure 9b) presents the last 11 data values (y) in blue, the 11 predicted values (*yp*) in red according to polynomial representation at ($p+1) = 6$, as well as the 11 predicted values (*yt*) in green according to autoregressive time-series representation of order 2.

It is not known a priori whether the data can be represented by a finite degree polynomial or a finite order time-series. The RMS error at $\left ({p+1 }\right)=5$ is 5142, while the RMS error at $q=2$ is 539. Clearly, the time-series representation is much more accurate. Also, the RMS error at $\left ({p+1 }\right)=6$ is 1711, much smaller than at lower $p$ values, but it is still much larger than the one from the time-series representation. Figure 9a) depicts all 61 data values ($y$) in blue, all 61 calculated values (*yp*) in red according to polynomial representation at $\left ({p+1 }\right)=6$ [the first 50 values are from the fit and the last 11 values are predictions], as well as all 61 calculated values (*yt*) in green according to autoregressive time-series representation of order 2 [the first 50 values are from the fit and the last 11 values are predictions]. To get a closer look at the predictions, Figure 9b) depicts the last 11 data values ($y$) in blue, the 11 predicted values (*yp*) in red according to polynomial representation at $\left ({p+1 }\right)=6$, as well as the 11 predicted values (*yt*) in green according to autoregressive time-series representation of order 2.

**FIGURE 10.**
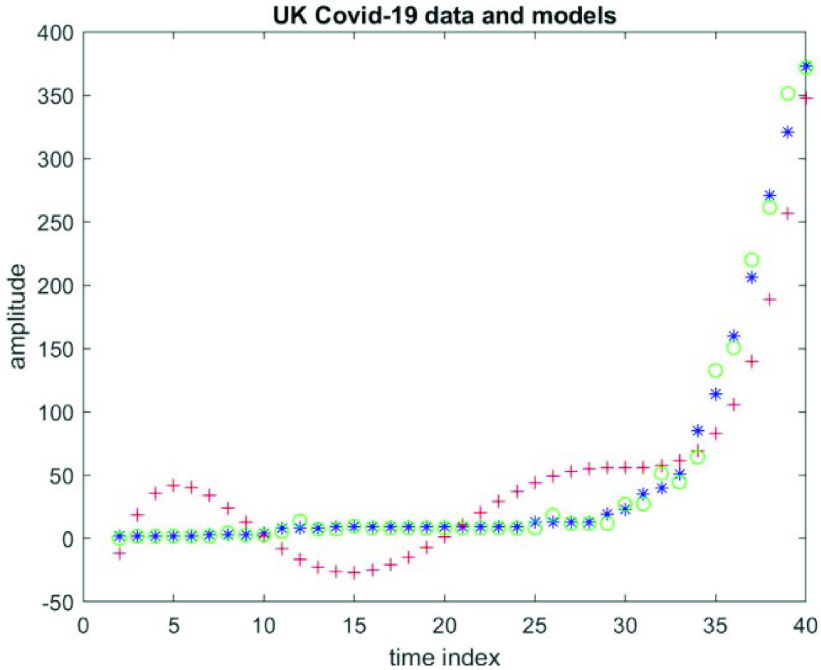
Cumulative daily confirmed cases of Covid-19 infections in *the* UK, covering the period from 31 January 2020 to 31 March 2020, Figure 10 plots data values (y) at $n=2, 3, \ldots, 40$ in blue, the corresponding fitted values (*yp*) in red according to polynomial representation at $(p+1) = 6$, as well as the corresponding fitted values (*yt*) in green according to autoregressive time-series representation of order 2.

To get a better idea of the fit (and not the predictions) Figure 10 plots data values $(y)$ at $n=2, 3, \mathrm { }\ldots, 40$ in blue, the corresponding fitted values (*yp*) in red according to polynomial representation at $\left ({p+1 }\right)=6$, as well as the corresponding fitted values (*yt*) in green according to autoregressive time-series representation of order 2.

It is clear that the polynomial representation picks up the trend of the later data values, but it completely fails for the first half of the data values. On the other hand, this autoregressive time-series of order 2 picks up the trend over the whole range of the data values. The results confirm that the UK Covid-19 data are significantly better described by an AR time-series of order 2 (less RMS error) than a finite degree polynomial of degree 5 (and others).

### F. Case VI

This is another example of using real data. The dataset represents cumulative daily confirmed cases of Covid-19 infections in the US. This dataset is publicly available [39]. On 04 April 2020 there were 25 data (i.e., N = 25) covering the period from 10 March 2020 to 03 April 2020. Thus $y(n)$ for $n=1:1:25$ represents the cumulative daily confirmed cases of Covid-19 infections in the US.

Of these 25 data, the first 15 data are used for estimating the free parameters and the last 10 data are used for forecasting. For a polynomial of the degree $p$, the first 15 data are used to estimate the $(p+1)$ coefficients of the polynomial using the Moore-Penrose inverse in much the same way as for Case V above. Using these estimated polynomial coefficients, all 25 data are calculated in a similar manner to Case V. The *YP* is a column vector of size $25\times 1$ and $YP=\left [{ yp\left ({1 }\right) yp\left ({2 }\right)\ldots yp\left ({25 }\right) }\right]^{T}$. $\left [{ yp\left ({1 }\right) yp\left ({2 }\right)\ldots yp\left ({15 }\right) }\right]^{T}$ came from the regression but $\left [{ yp\left ({16 }\right) yp\left ({17 }\right)\ldots yp\left ({25 }\right) }\right]^{T}$ are polynomial predictions for $\left [{ y\left ({16 }\right) y\left ({17 }\right)\ldots y\left ({25 }\right) }\right]^{T}$.

Similarly, for a time-series of order $q$, the first 15 data are used to estimate the $q$ coefficients of the time-series. Each of these data values depends on the coefficients and earlier data values. As all data values are error prone, the Total Least Squares, which takes account of errors in both the dependent and independent variables, is more appropriate than the ordinary Least Squares, which takes account of only errors in dependent variables and not in the independent variables. Using the $q$ estimated coefficients, all 25 data are calculated, i.e., $YT=\left [{ yt\left ({1 }\right) yt\left ({2 }\right)\ldots yt\left ({25 }\right) }\right]^{T}$. Of course, out of these 25 values, $\left [{ yt\left ({1 }\right) yt\left ({2 }\right)\ldots yt\left ({15 }\right) }\right]^{T}$ came from the regression and $\left [{ yt\left ({16 }\right) yt\left ({17 }\right)\ldots yt\left ({25 }\right) }\right]^{T}$ are time-series predictions for $\left [{ y\left ({16 }\right) y\left ({17 }\right)\ldots y\left ({25 }\right) }\right]^{T}$.

**FIGURE 11.**
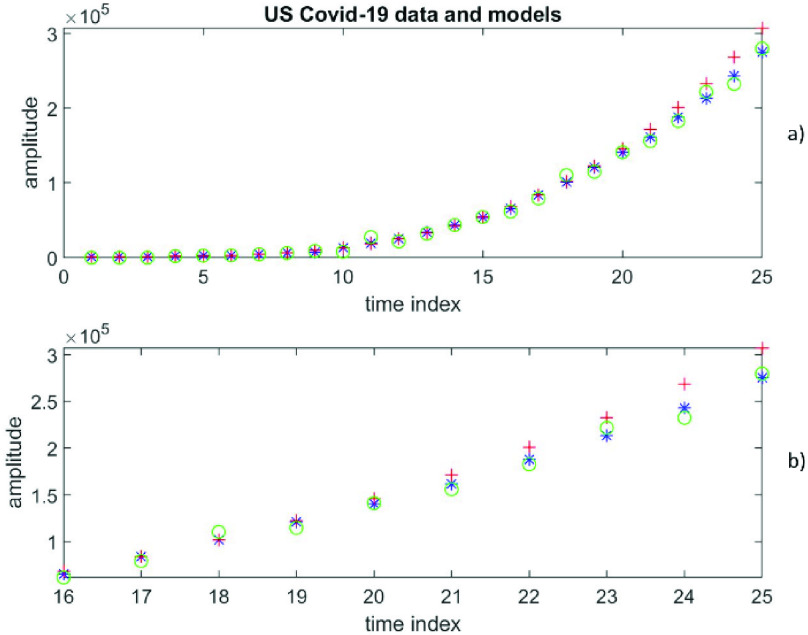
Cumulative daily confirmed cases of Covid-19 infections in the US, covering the period from 10 March 2020 to 03 April 2020, Figure 11a) depicts the 25 data values (y) in blue, 25 calculated values (*yp*) in red according to polynomial representation at $(p+1) = 4$ [the first 15 values are from the fit and the last 10 values are predictions], as well as 25 calculated values (*yt*) in green according to autoregressive time-series representation of order 2 [the first 15 values are from the fit and the last 10 values are predictions]. To get a closer look at the predictions, Figure 11b) shows the last 10 data values (y) in blue, the 10 predicted values (*yp*) in red from polynomial representation at $(p+1) = 4$, as well as the 10 predicted values (*yt*) in green from autoregressive time-series representation of order 3.

It is not known a priori whether the data can be represented by a finite degree polynomial or a finite order time-series. The RMS error at $\left ({p+1 }\right)=4$ is 15272, while the RMS error at $q=3$ is 6533. Figure 11a) depicts the 25 data values ($y$) in blue, 25 calculated values (*yp*) in red according to polynomial representation at $\left ({p+1 }\right)=4$ [the first 15 values are from the fit and the last 10 values are predictions], as well as 25 calculated values (*yt*) in green according to autoregressive time-series representation of order 2 [the first 15 values are from the fit and the last 10 values are predictions]. To get a closer look at the predictions, Figure 11b) shows the last 10 data values ($y$) in blue, the 10 predicted values (*yp*) in red according to polynomial representation at $\left ({p+1 }\right)=4$, as well as the 10 predicted values (*yt*) in green according to autoregressive time-series representation of order 3. RMS errors increase for other choices of $\left ({p+1 }\right)$ values. Clearly, the time-series representation is much more accurate.

**FIGURE 12.**
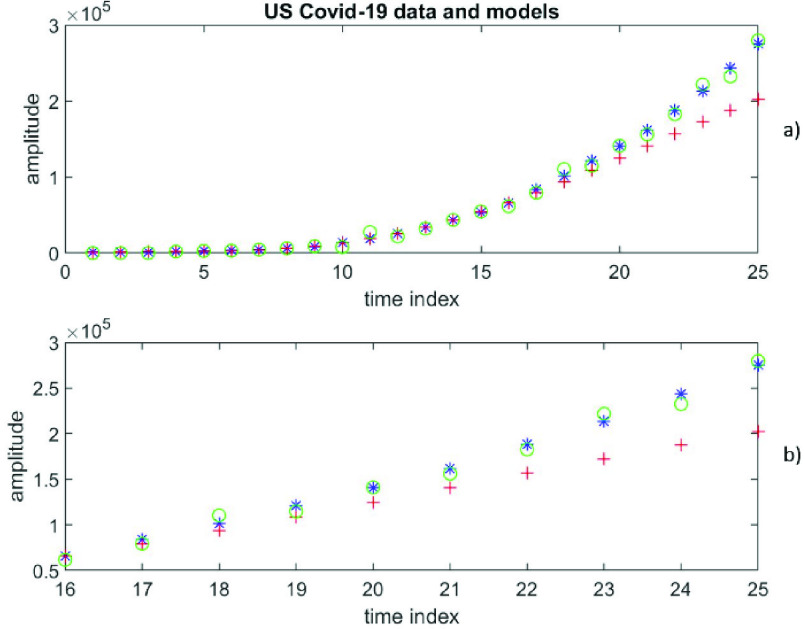
Daily confirmed cases of Covid-19 infections in the US, covering the period from 10 March 2020 to 03 April 2020, Figure 12a) and Figure 12b) are for $(p+1) = 5$; otherwise, they can be similarly described as in Figure 11, except for a different value of ($p+1$).

Looking for better results with a higher degree of polynomial, the RMS error at $\left ({p+1 }\right)=5$ is found to be 34692, which is significantly larger than the value from time-series representation at $q=2$. Figure 12a) and Figure 12b) for $\left ({p+1 }\right)=5$ can be similarly described as Figure 11 for $\left ({p+1 }\right)=4$. The results confirm that these US Covid-19 data are significantly better described by an AR time-series of order 3 than a finite degree polynomial of degree 3 (and others).

Table 2 provides a summary of these six cases. Data from a polynomial of finite degree can be represented equally well by a finite degree polynomial as well as a finite order time-series with specific integer coefficients, while data from other sources are represented significantly better by time-series representations. In many cases, finite order time-series can theoretically represent data from infinite order polynomials extremely well. Therefore, whenever the knowledge of the polynomial coefficients is not necessary in an application, one may choose to use time-series representation.

**TABLE 2:** Summary of Six Cases

Data source	Number of data	Degree of polynomial	Polynomial representation	Time-series representation	Comments
Case I Polynomial	35	3	At $\left ({p+1 }\right)=4$, RMS prediction error of 10^−12^.	At $q=4$, RMS prediction error of 10^−9^.	Both offer very low prediction errors.
Case II Sine wave	35	$\infty $	At $\left ({p+1 }\right)=1$, RMS prediction error of 3.8.	At $q=2$, RMS prediction error of 10^−15^.	Time-series offers significantly better prediction.
Case III Non-polynomial	35	$\infty $	At $\left ({p+1 }\right)=3$, RMS prediction error of 10^6^.	At $q=4$, RMS prediction error of 10^−8^.	Time-series offers significantly better prediction.
Case IV Inverse polynomial	35	$\infty $	At $\left ({p+1 }\right)=3$, RMS prediction error of 6.7.	At $q=7$, RMS prediction error of 10^−8^.	Time-series offers significantly better prediction.
Case V UK Covid-19 data	61	Unknown	At $\left ({p+1 }\right)=6$, RMS prediction error of 1711.	At $q=2$, RMS prediction error of 539.	Time-series offers much better prediction.
Case VI US Covid-19 data	25	Unknown	At $\left ({p+1 }\right)=4$, RMS prediction error of 15272.	At $q=3$, RMS prediction error of 6532.	Time-series offers much better prediction.

## V. Time-Series with Other Coefficients

It has been demonstrated in sections II and III that all data from polynomials of finite degree $q$ can be perfectly represented by a time-series of order $q$, if $\mu $ is not zero. The coefficients of such time-series are always integers of a specified form. Below are demonstrated what time-series with other forms of coefficients (either non-integers or integers of different forms) represent.

The equation (2) is called non-homogeneous if $\mu $ in equation (2) is not zero [40], [41]. Then equation (2) can be combined with its equivalent form

\begin{equation*} y\left ({n-1 }\right)= \sum \limits _{i=1}^{q} {a\left ({i }\right) y\left ({n-{1-i} }\right)+ \mu }\tag{34}\end{equation*}

to obtain (by replacing $\mu$)

\begin{equation*} y\left ({n }\right)= \sum \limits _{i=1}^{q+1} {\left ({a\left ({i }\right)-a\left ({i-1 }\right) }\right)y\left ({{n-i} }\right)}\tag{35}\end{equation*}

with $a\left ({0 }\right)\equiv -1$ and $a\left ({q+1 }\right)\equiv 0$. This is a homogeneous equation. The corresponding characteristics polynomial has $\left ({q+1 }\right)$ roots, i.e., $z\left ({1 }\right), z\left ({2 }\right), \ldots, z\left ({q+1 }\right)$. When these roots are distinct,

\begin{align*} y\left ({n }\right)&=b\left ({1 }\right)z\left ({1 }\right)^{n}+b\left ({2 }\right)z\left ({2 }\right)^{n}+\ldots \\& \qquad \qquad \qquad \qquad \qquad \qquad { + b\left ({q+1 }\right)z\left ({q }\right)^{n} } \tag{36}\end{align*}

On the other hand, when there are repeated roots, the solution is different. For only two repeated roots, e.g., $z\left ({1 }\right)=z\left ({2 }\right)=z$,

\begin{align*}&\hspace {-.5pc} y\left ({n }\right)\!=\!b\left ({1 }\right)z^{n}\!+\!b\left ({2 }\right)nz^{n} \!+ \!b\left ({3 }\right)z\left ({3 }\right)^{n}\!+\!\ldots \!+\!b\left ({q\!+\!1 }\right)z\left ({q }\right)^{n} \\ {}\tag{37}\end{align*}

Each of these two solutions in equations (36) and (37) describes polynomials of infinite degrees. Hence, finite order time-series with other forms of coefficients (either non-integers or integers of different forms) represent polynomials of infinite degrees.

### A. Example I

In case II above, $N$ data were generated from a sine wave

\begin{equation*} y\left ({n }\right)=sin(2\pi n/16), \quad \text {for} ~n=-17:1:17.\end{equation*}

Fitting $y\left ({n }\right)=a\left ({1 }\right)y\left ({n-1 }\right)+a\left ({2 }\right)y(n-2)$ to the first 4 data values, it was found that $a\left ({1 }\right)=1.8478$ and $a\left ({2 }\right)=-1.000$. These give rise to the characteristic polynomial of $z^{2}-1.8478 z+1=0$. The two roots are given by $z\left ({1 }\right)=0.9239+0.\mathrm {d}3827j$ and $z\left ({2 }\right)=0.9239-0.3827j$. As these two roots are distinct, the solution is given by $y\left ({n }\right)=b\left ({1 }\right)z\left ({1 }\right)^{n}+b\left ({2 }\right)z\left ({2 }\right)^{n}$. Since $y\left ({0 }\right)=0$, $b\left ({1 }\right)=-b\left ({2 }\right)$. Also, since $y\left ({4 }\right)=1$, $b\left ({1 }\right)=-0.5j$. So,

\begin{align*} y\left ({n }\right)=&-0.5j\left ({0.9239\!+\!0.3827j }\right)^{n}\!+\!0.5j\left ({0.9239\!-\!0.3827j }\right)^{n} \\=&\mathrm { }-0.5j \left ({\exp \left ({\frac {2\pi }{16} }\right) }\right)+ 0.5j \left ({\exp \left ({-\frac {2\pi }{16} }\right) }\right)^{n} \\=&sin(2\pi n/16)\end{align*}

This demonstrates how a time-series of order 2 can represent perfectly this sine wave which is a polynomial of infinite degree.

### B. Example II

Here $N$ data are generated from a non-polynomial

\begin{equation*} y\left ({n }\right)\!=\!1\!-\!2n\!-\!3n\left ({n-1 }\right)+\left ({0.5 }\right)^{n}, \quad \text {for}~n\!=\!-17:1:17.\end{equation*}

Fitting $y\left ({n }\right)=a\left ({1 }\right)y\left ({n-1 }\right)+a\left ({2 }\right)y\left ({n-2 }\right)+a\left ({3 }\right)\ast y\left ({n-3 }\right)+a\left ({4 }\right)y(n-4)$ to the first 8 data values, it is found that $a\left ({1 }\right)=3.5, a\left ({2 }\right)=-4.5, a\left ({3 }\right)=2.5$, and $a\left ({4 }\right)=-0.5$. These give rise to the characteristic polynomial of $z^{4}-3.5 \mathrm { }z^{3}+{4.5 z}^{2}-2.5 \mathrm { }z+0.5=0$. The four roots are given by $z\left ({1 }\right)=z\left ({2 }\right)=z\left ({3 }\right)=1=z$ and $z\left ({4 }\right)=0.5$. As these are three repeated roots, the solution is given by $y\left ({n }\right)=\left ({b\left ({1 }\right)+b\left ({2 }\right)n+b\left ({3 }\right)n\left ({n-1 }\right) }\right){(+1)}^{n}+b\left ({4 }\right)\left ({0.5 }\right)^{n}$. Using the first 8 data values, it can be shown that $b\left ({1 }\right)=1, b\left ({2 }\right)=-2, \mathrm { }b\left ({3 }\right)=-3$, and $b\left ({4 }\right)=1$. Therefore, $y\left ({n }\right)=1-2n-3n\left ({n-1 }\right)+\left ({0.5 }\right)^{n}$.

This is yet another example of how a finite order time-series (in this case of order 4) can represent perfectly this polynomial of infinite degree.

## VI. All-Pole Filters and Polynomials

In this section a connection between polynomials and all-pole filters is demonstrated.

### A. Polynomials and All-Pole Filters

It is well known that AR time-series models can be realised with all-pole filters. It has already been proven in Section III that all polynomials of finite degree of $q$ can be represented by AR time-series of order $q$ (as in equation (2)). Using z-transform, equation (2) can be written as

\begin{equation*} Y\left ({z }\right)= \mu /\left({1-\sum \limits _{i=1}^{q} {a\left ({i }\right) z^{-i}} }\right)\end{equation*}

and it has been proven in Section III that

\begin{align*} a\left ({i }\right)=\left ({-1 }\right)^{i+1}\left({\begin{array}{c}q\\ i \end{array}}\right)\end{align*}

for $i=1, 2, \ldots, \mathrm { }q$. So, the denominator polynomial can now be written as

\begin{align*}&\hspace {-0.5pc} 1- \sum \limits _{i=1}^{q} {\left ({-1 }\right)^{i+1}\left({\begin{array}{c}q\\ i \end{array}}\right)} z^{-i} \\[-2pt]& \qquad \qquad \qquad { 1+ \sum \limits _{i=1}^{q} {\left ({-1 }\right)^{i}\left({\begin{array}{c}q\\ i \end{array}}\right)} z^{-i}=\left ({1-z^{-1} }\right)^{q}} \end{align*}

Therefore, all polynomials of finite degree $q$ map onto $z=1$ on the z-plane by its $q$ repeated roots.

### B. Other Roots on the Unit Circle

All roots on the unit circle away from $z=-1$ and $z=1$ are complex. For a real-valued time-series, complex roots come in complex conjugate pairs. Consider just one such pair for illustration, i.e.,

$z\left ({1 }\right)=e^{-j\theta }$ and $z\left ({2 }\right)=e^{+j\theta }$. Thus, $y\left ({n }\right)=b\left ({1 }\right)z\left ({1 }\right)^{n}+b\left ({2 }\right)z\left ({2 }\right)^{n}=b\left ({1 }\right)e^{-j\theta n}+b\left ({2 }\right)e^{+j\theta n}$. Since $y\left ({n }\right)$ is real-valued, either $b\left ({1 }\right)=b(2) \equiv b$ and $y\left ({n }\right)=2b\cos \left ({\theta n }\right)$, or $b\left ({1 }\right)=-b\left ({2 }\right)\equiv -b/j$ and $y\left ({n }\right)=2b\sin \left ({\theta n }\right)$. Therefore, each pair of complex conjugates roots represent either a cosine or a sine, which can be described by an AR time-series of order 2 instead of a polynomial of infinite degree. The corresponding time-series coefficients are $a\left ({1 }\right)=2\cos \left ({\theta }\right)$ and $a\left ({2 }\right)=-1$.

### C. Other Complex Conjugate Roots Not on the Unit Circle

Again, for a real-valued time-series, complex roots come in complex conjugate pairs, consider just one pair for illustration, i.e., $z\left ({1 }\right)={\beta e}^{-j\theta }$ and $z\left ({2 }\right)=\beta e^{+j\theta }$, with $0< \beta < 1$. In this case, $y\left ({n }\right)=b\left ({1 }\right)z\left ({1 }\right)^{n}+b\left ({2 }\right)z\left ({2 }\right)^{n}=b\left ({1 }\right) \beta ^{n}e^{-j\theta n}+b\left ({2 }\right){\mathrm { }\beta ^{n}e}^{+j\theta n}$. Since $y\left ({n }\right)$ is real-valued, either $b\left ({1 }\right)=b(2) \equiv b$ and $y\left ({n }\right)=2b\beta ^{n}\cos \left ({\theta n }\right)$, or $b\left ({1 }\right)=b\left ({2 }\right)\equiv -b/j$ and $y\left ({n }\right)=2b\beta ^{n}\sin \left ({\theta n }\right)$. Therefore, each pair of complex conjugates roots represent either a damped cosine or a damped sine, which can be described by an AR time-series of order 2 instead of a polynomial of infinite degree. The corresponding time-series coefficients are $a\left ({1 }\right)=2\beta cos\left ({\theta }\right)$ and $a\left ({2 }\right)=-\beta ^{2}$.

### D. Real Roots Between −1 and +1 

Let $-1< z\left ({1 }\right),z\left ({2 }\right),z(3)< 1$ be the three distinct roots of the denominator polynomial. Then $y\left ({n }\right)=b\left ({1 }\right)z\left ({1 }\right)^{n}+b\left ({2 }\right)z\left ({2 }\right)^{n}+b\left ({3 }\right)z\left ({3 }\right)^{n}$. This can be described by an AR time-series of order 3 rather than a polynomial of infinite degree.

On the other hand if $-1< z\left ({1 }\right),z\left ({2 }\right),z(3)< 1$ be the three repeated roots of the denominator polynomial, i.e., $z\left ({1 }\right)=z\left ({2 }\right)=z(3) \equiv z$. In that case $y\left ({n }\right)=z^{n} \mathrm { }[b\left ({1 }\right)+b\left ({2 }\right)n+b\left ({3 }\right)n\left ({n-1 }\right)]$. This is another example of a finite order AR time-series representing data that requires a polynomial of infinite degree.

The two lessons are:

1) All polynomials of degree $q$ can be represented by an all-pole filter with $q$ repeated roots (or poles) at $z=+1$.
2) Data representable by finite order all-pole filters, whether they are from finite degree or infinite degree polynomials, can be described by a finite order AR time-series.

## VII. Conclusion

Two of the data modelling techniques are polynomial representation and time-series representation. In this paper, all theoretical studies to explore their connections and differences have been based on uniformly sampled data in the absence of errors. It has been proven that all data from an underlying polynomial model of finite degree $q$ as in equation (21) can be represented perfectly by either a polynomial of degree $q$ or an autoregressive time-series of order $q$ and a constant term. Also, it has been proven that all polynomials of degree $q$ can be described by the same set of time-series coefficients with the only possible difference being in the constant term $\mu $ as in equation (2). These time-series coefficients are integers of a specific form. It was also demonstrated that time-series with either non-integer coefficients or integer coefficients of not the specific form represent polynomials of infinite degree. Explorations, in four cases with generated data and in two cases with real data, demonstrated that, while finite degree polynomial and finite order time-series representations are equally good for data following finite degree polynomial forms, finite order autoregressive time-series representations offer significant advantages in modelling data from other sources. All polynomials of degree $q$ can be represented by an all-pole filter with $q$ repeated roots (or poles) at $z=+1$. Theoretically, all data representable by a finite order all-pole filter, whether they come from finite degree or infinite degree polynomials, can be described by a finite order AR time-series. If the values of polynomial coefficients are not necessary in an application, one may choose to use finite order time-series representations as they are more general than finite degree polynomial representations.
